# Binge eating among older women: prevalence rates and health correlates across three independent samples

**DOI:** 10.1186/s40337-021-00484-8

**Published:** 2021-10-19

**Authors:** Salomé Adelia Wilfred, Carolyn Black Becker, Kathryn E. Kanzler, Nicolas Musi, Sara E. Espinoza, Lisa Smith Kilpela

**Affiliations:** 1 Department of Psychology, University of Missouri-Kansas City, Kansas City, MO USA; 2 Department of Psychology, Trinity University, San Antonio, TX USA; 3 ReACH Center, UT Health San Antonio, San Antonio, TX USA; 4 Barshop Institute, UT Health San Antonio, San Antonio, TX USA; 5 South Texas VA Health System, Audie Murphy Veterans Hospital, San Antonio, TX USA

**Keywords:** Aging, Binge eating, Women’s health

## Abstract

**Background:**

Emerging research indicates that binge eating (BE; consuming unusually large amounts of food in one siting while feeling a loss of control) is prevalent among older women. Yet, health correlates of BE in older adult populations are poorly understood. The original study aimed to investigate BE prevalence, frequency, and health correlates in a sample of older adult women. Based on results from this first study, we then sought to replicate findings in two additional samples of older adult women from separate studies.

**Method:**

Using self-reported frequencies of BE from three separate samples of older women with very different demographics, we compared BE prevalence, frequency, and health correlates among older women. Study 1 (*N* = 185) includes data collected online (86% White; 59% overweight/obese status). Study 2 (*N* = 64) was conducted in person at a local food pantry (65% Hispanic; 47% household income < $10,000/year). Study 3 (*N* = 100) comprises data collected online (72% White; 50% Masters/Doctoral Degree).

**Results:**

Per DSM-5 frequency criterion of BE at least weekly, we found prevalence rates ranging from 19 to 26% across the three samples. Correlates of BE frequency included elevated negative mood, worry, BMI, and less nutritious food consumption.

**Conclusions:**

Across three very different samples in terms of race/ethnicity, education, food security status, measurements, and sampling methodology, we found fairly consistent rates of self-reported BE at least weekly (19–26%). Results suggest that BE is related to negative health indices among older women and support the need for more research in this population.

## Plain English summary

Historically viewed as problems of youth, eating disorders are understudied in older women. Yet, disordered eating, especially binge eating (BE; eating an unusually large amount of food with loss of control), appears to be more prevalent among older women than previously thought. Importantly, BE is associated with various health problems in the general population, but less is known about health outcomes related to BE in older populations. We compared prevalence rates of weekly BE measured in three separate samples of older women, as well as various health and wellness indices. We found fairly consistent rates of BE at least weekly (19–26%) across the three samples, and that BE frequency was related to negative health outcomes among older adult women. There is still much to be learned about BE among older adult populations. Future research is needed to better understand how specific age-related experiences may comprise risk factors for disordered eating among older women.

## Background

Nutrition pathology can significantly interfere with healthy aging. One form of nutrition pathology is disordered eating. Although clinical eating disorders and subclinical disordered eating are commonly viewed as problems of youth, emerging research indicates that older women indeed struggle with the spectrum of disordered eating [1, 2]. One of the most common forms of disordered eating among midlife and older women appears to be binge eating (BE) [3], which refers to recurrently consuming unusually large amounts of food in one siting while simultaneously feeling a loss of control, often resulting in significant distress [4]. BE is one of the core symptoms of binge eating disorder [4].

In the general population, binge eating disorder is associated with significant medical morbidity, including sleep problems, chronic pain, dyslipidemias, and metabolic and cardiac dysfunction [5, 6]. BE also increases risk for poorer micronutrient intake, as binge episodes typically involve consumption of foods high in sugar, fat, and salt [7, 8]. Frequent consumption of such foods and in large quantities can cause spikes in blood glucose [9] and research indicates that BE is linked prospectively with both type 2 diabetes and metabolic syndrome [10, 11]. Additionally, binge eating disorder is associated with higher levels of disability, psychosocial stress, and poorer quality of life [5, 6]. Importantly, BE also is closely associated with obesity and depression [12], which can amplify existing medical morbidities common among older adults. In older women, the health consequences of any form of disordered eating may be even more severe than seen in the general population, as older women have pre-existing risk for nutritional disorders (both over- and under-nutrition), sarcopenia, and frailty [13]. Despite this, older women historically have been excluded from BE research, leaving a major gap in understanding the risk, maintenance, and health consequences of BE among older women.

Eating disorder researchers have historically paid scant attention to older women. Despite this lack of research, numerous aging-related factors may contribute to elevating risk for BE in this population. Specifically, experiences related to the menopausal transition comprise risk for BE [3, 14]. For instance, a recent pilot study found that elevated daily estradiol, when progesterone was also elevated, was associated with BE episodes in perimenopausal women [14]. Sleep disturbances and negative affect, both of which are common experiences of women during the menopausal transition [15], also are well-established risk factors for BE [16, 17]. Additionally, psychosocial stressors, such as caregiving demands and interpersonal strain, may increase further risk for dysregulated eating behaviors, especially for women [18]. Indeed, preliminary research suggests that older women who experience life stressors such as divorce, marital conflicts, traumatic illness, empty nest syndrome, and loss of parents, siblings or children are at increased risk for onset of disordered eating [19].

Additionally, aging-related changes in body shape/appearance (e.g., increased adipose tissue, decreased muscle mass, increased body fat distribution towards the torso, and changes in skin coloration, firmness, and elasticity; [20–22] may also increase risk for disordered eating [3, 23]. These changes shift women’s bodies further away from the thin-young-ideal standard of female beauty in Western societies. This increased deviation from societal standards of female beauty, in turn, further increases risk for harmful comparisons of self to media representations and/or one’s younger body weight or shape [24]. Such upward body comparisons often lead to or exacerbate negative body image (also referred to as body dissatisfaction), which is a well-established and robust risk factor for BE [25].

Notably, recent research indicates that older women experience high levels of body dissatisfaction. For instance, Mangweth-Matzek et al. [2] found that over 60% of women age 60–70 years endorsed body dissatisfaction. Kilpela et al. [26] found that negative body image mediated the relation between BMI and both health/wellness behaviors as well as quality of life among women aged 50 and over. Thus, negative body image appears to be prevalent among older women and increase risk for both disordered eating and poorer health outcomes. In sum, early research investigating prevalence rates of BE among older women indicate that this pathology is more pervasive than previously thought. Yet, given that the aging process poses unique risk for women with regards to BE, there is still much to learn about both prevalence and health correlates of BE among older women.

As noted above, the intersection of aging and eating disorders is an emerging field of research. When broaching a new area of science, it is important that researchers are accountable and present well-informed findings, specifically when researching populations not often represented in research. The replication of research findings is one way to increase accountability, especially when presenting research findings in emerging domains.

Indeed, recent literature has documented a “replication crisis” in psychological science highlighting the importance of replicating findings in independent studies [27]. In their seminal paper, Open Science Collaboration set out to replicate 100 experiments; only 36% yielded a statistically significant effect in the replication effort. This outcome signaled a concerning lack of replication and raised significant questions regarding the validity of the original findings. In response to rising concerns about replication in psychological science and the paper by the Open Science Collaboration, Lindsay and editors at Psychological Science [28] identified three factors (referred to as the “troubling trio”) that appeared to contribute to lack of replication. These factors are, in terms coined by Lindsay: (1) “low statistical power,” (2) “a surprising finding,” and (3) “a *p* value of only slightly less than *p* = 0.05” [28]. According to Lindsay [28], any research findings that meet one or more of these criteria warrant efforts for replication.

### The present research

The original purpose of this research was to examine the prevalence of BE and health correlates in older women in a single study. However, the primary finding of this original study (hereafter referred to as the index study) met Lindsay’s criterion of a “surprising finding,” which is one of the “troubling trio” [28]. Specifically, the prevalence rate of weekly BE in the index study was higher than those seen in the limited existing research (e.g., [1–3]). This raised the specter of a spurious finding which led to the development of three specific aims for a series of studies presented in this paper. First, we sought to describe the frequency of binge eating in older women. Per DSM-5 diagnostic criteria for bulimia nervosa and binge eating disorder [4], which require binge eating once per week on average, we used the frequency criterion of weekly or more BE in our descriptions of prevalence rates within each sample. Second, we sought to identify the health behaviors correlated with binge eating in older women. Finally, we sought to determine if findings from the index study would replicate by comparing the findings of two additional samples of older women.

## Method

### Index study: general online sample of women

#### Participants

Participants were 185 women recruited online. Participants ranged from 60 to 83 years of age (*M* = 65.91, *SD* = 5.09); 86.4% identified as White and 2.2% endorsed Hispanic/Latina ethnicity (Table 1). Over one-third of participants (36.4%) reported a body mass index (BMI) greater than 30 (i.e., obese status), while 23.2% reported a BMI in the overweight range (25–29.9).

**Table 1:** Participant demographics and descriptive statistics for all three samples

	Study 1 (*N* = 185)	Study 2 (*N* = 64)	Study 3 (*N* = 100)
	% or *M* (SD)	%	% or *M* (SD)
Age	65.91 (5.09)	84.4% (Age 66–75) 15.6% (Age 76+)	60.57 (5.05)
Race/Ethnicity			
Black	9.2%	15.9%	16.8%
White	86.4%	14.3%	72.0%
Hispanic/Latinx	2.2%	65.1%	2.0%
Other/mixed	4.3%	4.8%	9.0%
Level of education			
Grade school or less	–	32.9%	0.0%
Some high school	–	15.6%	0.0%
High school diploma/GED	–	26.6%	5.0%
Some college/technical school	–	23.4%	16.8%
Bachelor’s degree	–	1.6%	18.8%
Some graduate school	–	0.0%	9.9%
Master’s degree	–	0.0%	34.7%
Doctoral degree	–	0.0%	14.9%
Annual household income			
Under $10,000	–	46.9%	–
$10,000-$40,000	–	37.5%	–
$40,000-$65,000	–	3.1%	–
BMI	28.29 (7.71)	–	26.62 (6.04)
Binge eating	26.5%	20.4%	19.0%
Compensatory behaviors			
Driven exercising	8.7%^a^	12.6%^b^	13.9%^b^
Self-induced vomiting	2.7%^a^	7.9%^b^	1.0%^b^
Laxatives or diuretics	2.7%^a^	11.0%^b^	3.0%^b^

Study 1 = General online sample; Study 2 = Women living with food insecurity; Study 3 = Women with high education levels; BMI = Body mass index; Binge Eating = binge eating at a frequency of weekly or more; Compensatory behaviors = behaviors endorsed for the purpose of weight/shape control

^a^ = Endorsed this behavior in the past week

^b^ = Endorsed this behavior in the past month

#### Procedure

The Institutional Review Board (IRB) of the senior author’s institution deemed this study as IRB-exempt (i.e., no more than minimal risk to study participants). Participants were recruited via email using personal and professional networks (i.e., non-randomized snowball sampling) and Amazon Mechanical Turk within the United States. We recruited women, aged 60 and over, to complete an online survey on health and wellness among older women. After providing informed consent, participants completed self-report questionnaires online.

### Measures

#### Demographic information

Participants reported age, height, weight, race and ethnicity. Although self-report is not optimal for assessing height and weight, research indicates that self-report weights are reasonably accurate [29] and we were limited to self-report due to the online nature of the study.

#### Binge eating

As our primary measure of BE, we used the VA-Binge Eating Screener (VA-BES; [30]), which asks: “On average, how often have you eaten extremely large amounts of food at one time and felt that your eating was out of control at that time?” Response options include: Never, < 1 time/week, 1 time/week, 2–4 times/week, and 5+ times/week. This measure demonstrated good psychometric properties [30].

#### Eating disorder (ED) symptoms

We assessed ED symptoms using the Eating Disorder Examination Questionnaire (EDE-Q Short; [31]), which is a 12-item version of the full EDE-Q [32]. The EDE-Q Short measures ED symptoms over the past week, using a 0–3 scale ranging from 0 to 7 days in the past week. Within this measure, one item asks about number of BE episodes in the past week. We did not isolate this item for any inferential statistics; however, we used self-reported BE frequency on this measure as an indicator of convergent validity participant responses to the primary measure of BE frequency (i.e., the VA-BES) to evaluate consistency of reporting. Past research demonstrated good internal consistency (Cronbach’s α = 0.913) and temporal stability (ICC = 0.93; *p* < 0.001; [31]). Internal consistency in the current sample was good (α = 0.86).

#### Negative affect

We used the 17-items of the fear, guilt and sadness subscales of the Positive and Negative Affect Scale-revised (PANAS-X; [33]) to measure negative affect over the past 3 weeks; higher scores indicate greater negative affect. This subscale has demonstrated good internal consistency in past research (current sample α = 0.95).

#### Consumption of nutritious foods

The two-item Eating Behaviors Questionnaire (EBQ; [34]) measured self-reported consumption of nutritious foods. Items inquire how often participants consciously tried to increase micronutrient density in meals and frequency of consuming fresh fruits and vegetables over the past week. The EBQ uses a 5-point Likert scale (1 = *consume at every meal*, 5 = *never*); items are summed, and higher scores indicate less consumption of nutritious foods. Internal consistency in this sample was adequate (α = 0.63).

## Results

We used descriptive statistics to evaluate BE prevalence and frequency in this sample. Due to the ordinal nature of BE frequency measure, we used Spearman’s rho to investigate correlations of BE frequency with BMI, ED symptoms, negative affect, and consumption of nutritious foods. Finally, we used a binomial logistic regression model to investigate the degree to which BE frequency contributed to risk for obesity status while controlling for age, race, and ethnicity. Regarding BE frequency and prevalence (Table 1), 48% of the sample reported never BE. Using the clinical frequency criterion of BE once per week or more, 26.5% of the sample reported BE at least weekly. As a secondary measure of BE frequency in this sample, we examined rates of BE using the BE item on the EDE-Q Short. One participant’s answer did not align with their response on the VA-BES, resulting in 25.5% of the sample endorsing at least one BE episode in the past week. BE frequency was correlated with higher BMI (⍴ = 0.40, *p* < 0.01), more ED symptoms (⍴ = 0.65, *p* < 0.001), greater negative affect (⍴ = 0.43, *p* < 0.01), and less frequent consumption of nutritious foods (⍴ = 0.16, *p* < 0.05). Finally, BE frequency contributed significant risk for obese status based on self-reported BMI (*OR* = 2.08; 95% CI [1.52; 2.82], *p* < 0.001). Age, race, and ethnicity categories were non-significant in the model.

### Study 2 (replication): sample living with food insecurity

#### Participants

This sample consisted of 64 older women (ages 66+) who were clients at food pantries of the local food bank who participated in a larger study of individuals living with food insecurity [35]. Age was only captured in clusters; thus, only women who selected the “age 66 or over” option were included in this study. Younger participants and men were not included. The majority of participants identified as Hispanic (65.1%; Table 1). Over a third (39.1%) reported disabled status, and 48.4% had less than a high school education or equivalent (GED). Almost half (46.9%) reported a household annual income of less than $10,000/year. Access to current health data is often low among individuals living with extreme poverty; scales are considered luxury items and often are not in the home [35]. Thus, requesting current height and weight in this sample was likely to elicit inaccurate data. Therefore, we do not have BMI data in this sample.

#### Procedure

This study received IRB approval and was run in collaboration with the local food bank. See Becker et al. [35] for details regarding the full study and community partnership. All questionnaires were available in either English or Spanish. Following informed consent, participants completed questionnaires in person; undergraduate research assistants facilitated reading as needed (i.e., read questions aloud in language of choice) and answered questions. Participants received a $5 gift card to a local grocery store as compensation.

### Measures

Consistent with working with a marginalized, low-education population and incorporating a socially conscious lens [36, 37], we evaluated survey complexity. Guided by a colleague with extensive experience working with marginalized populations, we employed best practices delineated by Stonewall and colleagues [38] to evaluate and modify questionnaire language. We modified or removed items based on reading level or that may impact comprehension (for detailed rationale and procedure for measures modification, see Becker et al. [35]). This process ensured that our survey used appropriate language in order to be inclusive and culturally sensitive [36].

#### Food insecurity

To assess severity of food insecurity, we used the Radimer Cornell Food Insecurity Measure (RCFIM: [39, 40]). This measure uses a Likert scale, and global scores indicate level of food insecurity: (1) not food insecure (i.e., did not meet criteria for food. insecurity); (2) household food insecurity (i.e., anxiety about food, eating the same thing repeatedly due to lack of resources, and food running out but no one going hungry in the home); (3) individual food insecurity (i.e., adult reports being hungry at times because they lack food); and (4) child hunger food insecurity (i.e., adult reports inability to adequately feed their children secondary to lack of resources). For this study, we only included participants in the two highest levels of food insecurity (individual and child hunger), as these are the most severe. Internal consistency was good in our sample (Cronbach’s α = 0.92).

#### Binge eating

We used the BE item from the Eating Disorder Diagnostic Scale for DSM 5 (EDDS-5) to assess frequency of BE over the past month [41]. The EDDS is a brief self-report measure designed to assess the spectrum of EDs. The BE item asks, “How many times in the past month (30–31 days) on average have you eaten an unusually large amount of food and experienced a loss of control?” A report of ≥ 4 times in the past month equated to weekly binge eating.

#### Internalized weight stigma

We used the Weight Self-Stigma Questionnaire (WSSQ) to assess internalization of weight bias [42]. Items are rated on a 7- point Likert scale (1 = *strongly disagree* to 7 = *strongly agree*), and participants rate their level of agreement with explicit weight bias statements (e.g., “I became fat because I’m a weak person” and “People think that I am to blame for being fat.”). Scores are summed for a total score and higher scores indicate greater self-stigma; internal consistency was excellent (α = 0.97).

#### Anxiety

We used eight items from the Penn State Worry Questionnaire (PSWQ; [43]) to assess anxiety/worry. Items are rated on a 5-point Likert scale (1 = *not at all typical of me* to 5 = *very typical of me*); higher scores indicate more anxiety/worry. Internal consistency for this version of the PSWQ in the current sample was excellent (α = 0.95).

## Results

Similar to the index study, we used descriptive statistics to evaluate BE prevalence and frequency in this replication sample. We used bivariate correlations of BE frequency with anxiety and internalized weight stigma. Finally, we examined rates of unhealthy weight control behaviors (e.g., self-induced vomiting, use of laxatives/diuretics for weight control) in this sample. Regarding BE prevalence, 20.4% of this sample reported BE on a weekly or more basis in the past month. Frequency of BE was positively correlated with higher anxiety (*r* = 0.37, *p* = 0.008) and internalized weight stigma (*r* = 0.42, *p* = 0.002). Within this sample of older women living with significant food insecurity, 7.9% of women reported self-induced vomiting, and 11% reported use of laxative or diuretics for weight control in the past month.

### Study 3 (replication): sample with high education levels

#### Participants

Participants were 100 women recruited online for a larger study of body image in a diverse sample of adult women. Participant ages ranged from 55–79 years (*M* = 60.57, *SD* = 5.05) and reported a mean body mass index of 26.62 (*SD* = 6.04). The majority (72.0%) self-identified as White; 2.0% identified as Hispanic/Latina, and 72% were currently married (Table 1). Notably, 50% of women in this sample had a Masters or Doctoral degree.

#### Procedure

IRB approval was granted for this study and participants were recruited via email, social media, personal and professional networks, and by word of mouth. Recruitment emails described the study as exploring body image and wellness in a diverse population of adult women. All emails and posts requested that women forward the study invitation to their own social and professional networks (i.e., snowball sampling). After providing informed consent, participants completed self-report questionnaires online and had the option to provide their email address to enter a raffle for a $200 Amazon gift card.

### Measures

#### Binge eating

We assessed BE with the diagnostic items of the EDE-Q [32], which is a self-report measure of eating behaviors and attitudes over the past 28 days. The BE items inquire about frequency of BE episodes over the past 28 days. A report of ≥ 4 times in the past four weeks (28 days) equated to weekly BE.

#### BMI

Participants self-reported their height and weight in order to calculate BMI.

#### Depressive symptoms

We used the Beck Depression Inventory- II (BDI-II; [44]) to measure depressive symptoms. Due to liability in assessing suicidality anonymously and via online survey, we removed the suicidality item from the BDI-II. Thus, our final measure included 20 items and had good internal validity in the current sample (α = 0.85).

#### Body shame

We administered the Shame subscale of the Objectified Body Consciousness Scale (OBCS; [45]). This subscale includes eight items rated from 1 (*strongly disagree*) to 7 (*strongly agree*), with a “not applicable” option for items that do not apply. Higher scores indicate greater shame, and 25% or more of items NA or missing qualifies as missing overall score. In the present sample, internal consistency was good (α = 0.85).

## Results

Similar to the previous studies, we used descriptive statistics to evaluate BE prevalence and frequency in this second replication sample. We used bivariate correlations of BE frequency with BMI, depressive symptoms, and body shame. Finally, we used a binomial logistic regression model to investigate the degree to which BE frequency contributed to risk for obesity status while controlling for age, race, and ethnicity. In this sample of women with high education levels, 19% reported BE weekly or more in the past month. BE frequency was positively correlated with greater depressive symptoms (*r* = 0.36,* p* < 0.001) and higher body shame (*r* = 0.44,* p* < 0.001), but was not correlated with BMI (*r* = 0.20, *p* = 0.075). Finally, BE frequency contributed a small, but statistically significant risk for obesity status (*OR* = 1.12; 95% CI [1.00, 1.24], *p* = 0.04). Neither age nor race/ethnicity were significant in the model.

## Discussion

The index study in this series of studies was designed to examine the frequency, prevalence, and health correlates of binge eating (BE) in a single sample of older women aged 60 and over. Findings from this index study indicated that roughly 26% of older women in our sample reported experiencing BE at a frequency of weekly or more in the past month. Notably, the clinical frequency criterion for a diagnosis of binge eating disorder or bulimia nervosa is BE weekly or more for at least 3 months [4]. Prior research, however, identified lower prevalence rates of weekly BE among midlife and older adult women. For instance, 11% of women in midlife reported regular BE as assessed by self-report with the Bulimia Test-Revised (BULIT-R; [12]). Using a non-validated self-report measure inquiring about frequency of BE episodes, 3.5% of women aged 50+ reported weekly BE [46]. Using a similar assessment measure, a sample of women aged 60–70 years, 3.8% met criteria for an ED, while another 4.4% endorsed at least one core symptom of an ED [2].

Notably, the vast majority of research examining prevalence rates and types of eating disordered behaviors among older adults used self-report measures, rather than gold standard clinical interviews (e.g., the Eating Disorders Examination; [47]). In one study that utilized a structured clinical interview, 5.6% of women aged 65–94 reported BE episodes in the past month, with mean frequency of 8 episodes/month [1]. Thus, prevalence rates of disordered eating documented in the literature ranged from roughly 4–11% and are smaller than our findings.

Of note, 59.6% of participants in the index study met BMI classification criteria for overweight (i.e., 25 < BMI < 30) or obese status (i.e., BMI > 30). To put this in context, past research investigating BE prevalence in obese patients presenting for weight loss treatment has documented clinically relevant BE at approximately 30% [48–50]. Thus, our observed prevalence rate for weekly BE in the index study was slightly lower than data from general adult patients (i.e., not older adults) presenting for weight loss programs. Our sample was neither a treatment-seeking sample, nor did we conduct targeted recruitment for clinical populations. Thus, the higher prevalence of overweight/obesity may have played a role in the elevated rates of BE in this first sample, as compared to past research and to the other two studies.

Because the prevalence rate of weekly or more BE from this index study was surprisingly high (i.e., a “surprising finding” per Lindsay; [28]), we were concerned about a spurious finding. Specifically, our findings fell into one construct of the “troubling trio” [28] in the replication crisis in psychological science, which raised concerns regarding replicability. Per recommendations from Lindsay [28], such results warrant efforts for replication. Therefore, we sought to replicate our index study results using existing data from two other samples of older adult women that included self-reported BE frequency. Results from both replications using data from two additional independent studies—with notably different demographics—were largely consistent with the index study.

We propose that the combined results suggest that BE rates in older women may be higher than previously thought and are deserving of further research. While these data do not examine the full diagnostic criteria for binge eating disorder, findings across the three studies provide preliminary data supporting the need for more research regarding eating disorders among older adults. The primary strength of the three studies, when viewed collectively, is the ability to refute the argument that the primary findings of the index study (i.e., BE prevalence and frequency) were artificially inflated due to one or more of the following three factors: (1) sampling biases secondary to recruitment strategy; (2) measurement error (i.e., risk of inherent response biases within one measure); and (3) participant demographics (i.e., limited generalizability). Regarding the first concern of sampling bias, two of the three studies used different types of internet sampling (i.e., snowball sampling, social media, and MTurk), whereas one used in-person recruitment and data collection. Despite the different sampling methods (internet versus in-person/community), prevalence rates of BE were similar across all three studies, suggesting the rates were not unique to one sampling method.

The second potential concern pertains to the way in which BE was assessed. For instance, it could be argued that the primary measurement strategy of the index study (i.e., the VA-BES) was flawed and resulted in inflated estimates of BE in the index sample. However, we used a total of four validated measures of BE across the three studies and findings remained remarkably consistent. Therefore, our findings regarding prevalence rates of BE were not simply due to measurement error. Of note, prior research utilized both validated and non-validated self-report instruments to assess rates of current binge eating among midlife and older women; only one study used a clinical interview.

Finally, one could argue, based on the index study alone, that the representativeness of the index sample in terms of sample characteristics (i.e., participant demographics) led to elevated assessment of BE. This supposition, however, does not appear to hold water when the nature of the three samples are compared. For instance, the community sample collected at local food pantries was predominantly Hispanic (65%), predominantly very low SES (47% reported an annual household income < $10,000/year) and living with significant food insecurity. Moreover, nearly 50% reported less than a high school degree or equivalent, and nearly 40% reported disabled status. In contrast, both of the internet samples were majority Non-Hispanic White, and one internet sample comprised women with high levels of education (50% Masters or Doctoral Degree). In summary, we found fairly consistent rates of self-reported BE at least weekly (19–26%) across three independent and very different samples in terms of race/ethnicity, education, food security status, measurements used, and data collection methods.

In addition to a high prevalence rate of weekly BE across the three samples, correlational analyses indicated that BE frequency was related to poorer psychological and physical health indices. Specifically, higher BE frequency was positively correlated with greater depression and negative affect, body shame, worry, and internalized weight stigma. Additionally, BE frequency was negatively correlated with frequency of consuming nutrient-dense foods. Lastly, results regarding BMI were mixed across two samples. In our index study, BE frequency was positively correlated with BMI, while this relation was nonsignificant in the highly educated sample. However, in both samples BE frequency increased risk for obesity status in our binomial logistic regression models. Thus, further investigation into obesity risk as a factor BE among diverse samples of older women is warranted.

Correlational findings from these three studies align with previous research on correlates of BE in younger samples. For instance, BE is consistently linked with depressive symptoms [12] and elevated BMI [51, 52] in prior research. Our correlational findings in this sample of older women are consistent with BE correlates observed in previous BE research conducted with more well-investigated samples (i.e., younger adults). In other words, expected BE and health correlates previously established hold true in this newer population of older adult women, which provides confidence in our interpretation of these data. Finally, these data extend prior research by documenting additional negative health correlates (e.g., internalized weight stigma, worry, and consumption of nutritious foods) of BE in older adult women.

It is important to note that we did not assess the full criteria for a diagnosis of binge eating disorder, per the DSM-5 [4]. Although women across these three separate studies reported BE weekly, this was only assessed in the past month. Additionally, we did not collect information on the emotional/cognitive criteria for binge eating disorder; rather, we only assessed the frequency of the behavior in the past month. Therefore, these findings may reflect a lower level of BE pathology severity in comparison to clinical-level binge eating disorder. Alternatively, our data support the contention that regular BE, regardless of full diagnostic status, is associated with poorer health constructs (e.g., depression/negative affect, less frequent consumption of micronutrients, body shame, worry, internalized weight stigma). Future research is needed to disentangle the potential health impacts of BE behaviors versus clinical threshold binge eating disorder among older adult populations.

There are numerous limitations to the current studies. First, all studies used self-report measures exclusively. Thus, it is entirely possible that results from self-report measures showed a greater frequency of BE as compared to those assessed by structured clinical interview. Further, we did not have objective measures of BMI nor clinical interviews to further explore clinical constructs. Second, although the three studies used different sampling methods, two of the three studies comprised minimal racial/ethnic diversity of participants and were online samples. Future research should use purposeful sampling methodology in order to evaluate more diverse samples of older adult women and therefore enhance generalizability of findings. Additionally, analyses were cross-sectional and correlative in nature, therefore we are unable to evaluate causality among variables within each study. Future research should include larger sample sizes, incorporate clinical interviews, and utilize longitudinal methodology designed to investigate causality and chronological outcomes. Lastly, future studies should assess the full diagnostic criteria for binge eating disorder, in order to better estimate the prevalence of this pathology among older adult women.

## Conclusions

In sum, findings indicated relatively stable rates of weekly or more BE (19–26%) among three independent and very different samples of older adult women. The rates observed across these three samples are higher than those documented in prior research and in younger samples, suggesting that older adult women may be at elevated risk for BE, yet further research is needed to examine the full diagnostic criteria for binge eating disorder. Correlations indicated that BE frequency is linked with negative health constructs, including depression and negative affect, body shame, worry, and internalized weight stigma. Longitudinal research is needed to better investigate the chronological effects of BE among older adult samples, as well as objective or interview methods of data collection.

## Data Availability

The datasets used and/or analyzed during the current study are available from the corresponding author on reasonable request.

## Abbreviations

BE: Binge eating
BMI: Body mass index
ED: Eating disorder
DSM-5: Diagnostic and Statistical Manual for Mental Disorders, 5th edition
IBR: Institutional Review Board
VA-BES: Veterans Affairs-Binge Eating Screener
EDE-Q: Eating Disorders Examination-Questionnaire
PANAS-X: Positive and Negative Affect Schedule
EBQ: Eating Behaviors Questionnaire
RCFIM: Radimer Cornell Food Insecurity Measure
EDDS-5: Eating Disorders Diagnostic Scale for DSM-5
WSSQ: Weight Self Stigma Questionnaire
PSWQ: Penn State Worry Questionnaire
BDI-II: Beck Depression Inventory-II
OBCS: Objectified Body Consciousness Scale
SES: Socioeconomic status
